# The Metamorphosis of the Hero: Principles, Processes, and Purpose

**DOI:** 10.3389/fpsyg.2019.00606

**Published:** 2019-03-21

**Authors:** Scott T. Allison, George R. Goethals, Allyson R. Marrinan, Owen M. Parker, Smaragda P. Spyrou, Madison Stein

**Affiliations:** Department of Psychology, University of Richmond, Richmond, VA, United States

**Keywords:** hero, heroic transformation, hero’s journey, heroic development, human development

## Abstract

This article examines the phenomenon of heroic metamorphosis: what it is, how it unfolds, and why it is important. First, we describe six types of transformation of the hero: mental, moral, emotional, spiritual, physical, and motivational. We then argue that these metamorphoses serve five functions: they foster developmental growth, promote healing, cultivate social unity, advance society, and deepen cosmic understanding. Internal and external sources of transformation are discussed, with emphasis on the importance of mentorship in producing metamorphic growth. Next we describe the three arcs of heroic transformation: egocentricity to sociocentricity, dependence to autonomy, and stagnation to growth. We then discuss three activities that promote heroic metamorphosis as well as those that hinder it. Implications for research on human growth and development are discussed.

## Introduction

One of the most revered deities in Hinduism is Ganesha, a god symbolizing great wisdom and enlightenment. Ganesha’s most striking attribute is his unusual appearance. In images throughout India and southeast Asia, he is shown to be a man with an ordinary human body and the head of an elephant. According to legend, when Ganesha was a boy, he behaved foolishly in preventing his father Shiva from entering his own home. Shiva realized that his son needed an entirely new way of thinking, a fresh way of seeing the world. To achieve this aim, Shiva cut off Ganesha’s human head and replaced it with that of an elephant, an animal representing unmatched wisdom, intelligence, reflection, and listening. Ganesha was transformed from a naïve boy operating with little conscious awareness into a strong, wise, and fully awakened individual.

This article is about how people undergo dramatic, positive change. We focus on heroic metamorphosis – what it is, how it comes about, and why it’s important. Unlike Ganesha, one need not undergo dramatic physical change to experience heroic transformation. One must engage in any of three types of activities that we describe in this article: (1) training regimens, (2) spiritual practices, and (3) the hero’s journey. Anyone who transforms as a result of these activities emerges a brand-new person, a much-improved version of one’s previous self. Metamorphosis and transformation are both defined as “changing form,” a process that precisely describes the massive alteration undergone by Ganesha. Having undergone the hero’s journey as the pathway to transformation, Ganesha sees the world with greater clarity and insight. The hero’s journey inevitably involves setback, suffering, and a death of some type. What dies is usually the former self, the untransformed version of oneself that sees the world “through a glass darkly” (Bergman, 1961). Ganesha’s decapitation happens to us all metaphorically; the journey marks the death of a narrow, immature way of seeing the world and the birth of a wider, more enlightened way of viewing life.

## Overview of Heroic Metamorphosis

Metamorphic change pervades the natural world, from the changing of the seasons to biological growth and decay (Wade, 1998; Allison, 2015; Efthimiou, 2015). The universe itself is subject to immense transformation on both a microscopic scale as well as a *trans*-universal scale. Biological cells grow, mutate, and die, and on a much grander scale the galaxies of the universe are in a constant state of flux. Darwinian theory portrays all of life as engaged in an inescapable struggle to survive in response to ever-changing circumstances. Life presents an ultimatum to all organisms: change as all phenomena in the universe must change, or fall.

Heroic transformation appears to be a prized and universal phenomenon that is cherished and encouraged in all human societies (Allison and Goethals, 2017; Efthimiou and Franco, 2017; Efthimiou et al., 2018a,b). Surprisingly, until the past decade there has been almost no scholarship on the topic of heroic transformation. Two early seminal works in psychology offered hints about the processes involved in dramatic change and growth in human beings. In 1902, William James addressed the topic of spiritual conversion in his classic volume, *The Varieties of Religious Experience*. These conversion experiences bear a striking similarity to descriptions of the hero’s transformation as reported by famed mythologist Campbell (1949). These experiences included feelings of peace, clarity, union with all of humanity, newness, happiness, generosity, and being part of something bigger than oneself. James emphasized the pragmatic side of religious conversion, noting that the mere belief and trust in a deity could bring about significant positive change independent of whether the deity actually exists. This pragmatic side of spirituality is emphasized today by Thich Nhat Hanh, who observes that transformation as a result of following Buddhist practices can occur in the absence of a belief in a supreme being. Millions of Buddhists have enjoyed the transformative benefits of religion described by James simply by practicing the four noble truths and the noble eightfold path (Hanh, 1999, p. 170).

The second early psychological treatment of human transformation was published in 1905 by Sigmund Freud. His *Three Essays on the Theory of Sexuality* described life-altering transformative stages in childhood involving oral, anal, phallic, and latent developmental patterns. None of these changes were particularly “heroic” but they did underscore Freud’s belief in the inevitability of immense psychological change. Although Freud suggested that people tend to resist change in adulthood, several subsequent schools of psychological thought have since proposed mechanisms for transformative change throughout the human lifespan. Humanistic theories, in particular, have embraced the idea that humans are capable of a long-term transformation into self-actualized individuals (e.g., Maslow, 1943). Developmental psychologists have also proposed models of transformative growth throughout human life (e.g., Erikson, 1994). Recent theories of self-processes portray humans as open to change and growth under some conditions (Sedikides and Hepper, 2009) but resistant under others (Swann, 2012). In the present day, positive psychologists are uncovering key mechanisms underlying healthy transformative growth in humans (Lopez and Snyder, 2011; Seligman, 2011).

An important source of transformation resides in tales of heroism told and re-told to countless generations throughout the ages. These mythologies reflect humanity’s longing for transformative growth, and they are packed with wisdom and inspiration (Allison and Goethals, 2014). Just reading, hearing, or observing stories of heroism can stir us and transform us.

According to Campbell (2004, p. xvi), these hero tales “provide a field in which you can locate yourself” and they “carry the individual through the stages of life” (p. 9). The resultant transformations seen in heroic stories “are infinite in their revelation” (Campbell, 1988, p. 183). Rank (1909, p. 153) observed that “everyone is a hero in birth, where he undergoes a tremendous psychological as well as physical transformation, from the condition of a little water creature living in a realm of amniotic fluid into an air-breathing mammal.” This transformation at birth foreshadows a lifetime of transformative journeys for human beings.

According to Allison and Goethals (2013, 2017), hero stories reveal three different targets of heroic transformation: *setting, self*, and *society*. These three loci of transformations parallel Campbell’s (1949) three major stages of the hero’s journey: departure (or separation), initiation, and return. The departure from the hero’s familiar world represents a transformation of one’s normal, safe environment; the initiation stage is awash with challenge, suffering, mentoring, and transformative growth; and the final stage of return represents the hero’s opportunity to use her newfound gifts to transform the world. The sequence of these stages is critical, with each transformation essential for producing the next one.

Without a change in setting, the hero cannot change herself, and without a change in herself, the hero cannot change the world. Our focus here is on the hero’s transformation of the self, but this link in the chain necessarily requires some consideration of the links preceding and following it. The mythic hero must be cast out of her familiar world and into a different world, otherwise there can be no departure from her status quo. Once transformed, the hero must use her newly enriched state to better the world, otherwise the hero’s transformation lacks social significance.

The hero’s transformation plays a pivotal role in her ability to achieve her objectives on the journey. During the quest, “ineffable realizations are experienced” and “things that before had been mysterious are now fully understood” (Campbell, 1972, p. 219). The ineffability of these new insights stems from their unconscious origins. Jungian principles of the collective unconscious form the basis of Campbell’s theorizing about hero mythology. Le Grice (2013, p. 153) notes that “myths are expressions of the imagination, shaped by the archetypal dynamics of the psyche.” As such, the many recurring elements of the mythic hero’s journey have their “inner, psychological correlates” (Campbell, 1972, p. 153). The hero’s journey is packed with social symbols and motifs that connect the hero to her deeper self, and these unconscious images must be encountered, and conflicts with them must be resolved, to bring about transformation (Campbell, 2004). Overall, the hero’s outer journey is a representation of an inner, psychological journey that involves “leaving one condition and finding the source of life to bring you forth into a richer or mature condition” (Campbell, 1988, p. 152).

Allison and Smith (2015) identified five types of heroic transformation: physical, emotional, spiritual, mental, and moral. A sixth type, motivational transformation, was later proposed by Allison and Goethals (2017). These six transformation types span two broad categories: physical transformation, which we call *transmutation*, and psychological transformation, which we call *enlightenment*. Physical transmutations are endemic to ancient mythologies that featured transforming humans into stars, statues, and animals. Today, transmutation pervades superhero tales of ordinary people succumbing to industrial accidents and spider bites that physically transform them into superheroes and supervillains. These ancient and modern tales of transmutation offer symbolism of the hidden powers residing within each of us, powers that emerge only after dramatic situations coax them out of hibernation. Efthimiou (2015, 2017), Franco et al. (2016), and Efthimiou and Allison (2017) have written at length about the power and potential of biological transmutation to change the world. The phenomenon of *neurogenesis* refers to the development of new brain cells in the hippocampus through exercise, diet, meditation, and learning. This transmutative healing and growing can occur even after catastrophic brain trauma. Efthimiou (2017) describes many examples of transmutation occurring as a result of *regeneration* or *restoration* processes that refer to an organism’s ability to grow, heal, and re-create itself.

Epigenetic changes in DNA and the science of human limb regeneration are two examples of modern day heroic transmutations (Efthimiou, 2015).

The other five types of heroic transformation – moral, mental, emotional, spiritual, and motivational – comprise the second broad category of transformation that we call enlightenment. Emotional transformations refer to “changes of the heart” (Allison and Smith, 2015, p. 23) involving growth in empathic concern for others; we call this transformation *compassion*.

Spiritual transformations refer to changes in belief systems about the spiritual world and about the workings of life, the world, and the universe; we call this change *transcendence*. Mental transformations refer to leaps in intellectual growth and significant increases in illuminating insights about oneself and others; we label this *wisdom*. Moral transformations occur when heroes undergo a dramatic shift from immorality to morality; we call this *redemption*. Finally, a motivational transformation refers to a complete shift in one’s purpose or perceived direction in life; we label this change a *calling* (see also Dik et al., 2017).

## Purpose of the Hero’s Transformation

The purpose of the hero’s journey is to provide a context or blueprint for human metamorphosis. Why do we need such life-changing growth? Allison and Setterberg (2016) argue that people are born “incomplete” psychologically and will remain incomplete until they encounter challenges that produce suffering and require sacrifice to resolve. Transcending life’s challenges enables the hero to “undergo a truly heroic transformation of consciousness,” requiring them “to think a different way” (Campbell, 1988, p. 155). This shift offers a new “map or picture of the universe and allows us to see ourselves in relationship to nature” (Campbell, 1991, p. 56). Buddhist traditions and twelve-step programs of recovery refer to transformation as an awakening. Using similar language, Campbell (2004, p. 12) described the function of the journey as a necessary voyage designed to “wake you up.” The long-term survival of the human race may depend on such an awakening, as it becomes increasingly clear that the unawakened, pre-transformed state is unsustainable at the collective level. As individuals, transformation is necessary for our psychological, emotional, social, and spiritual well-being. Collectively, the survival of our planet may depend on broader, enlightened thinking from leaders who must be transformed themselves if they are to make wise decisions about human rights, climate change, peace and war, healthcare, education, and myriad other pressing issues. Nearly 50 years ago, Heschel (1973) opined that “the predicament of contemporary man is grave. We seem to be destined either for *a new mutation* or for destruction” (p. 176, italics added).

Allison and Goethals (2017) propose five reasons why transformation is such a key element in the hero’s journey, and why it is essential for promoting our own and others’ welfare. First, transformations foster developmental growth. Early human societies understood the usefulness of initiation rituals in promoting the transition from childhood to adulthood (van Gennep, 1909). A number of scholars, including Campbell, have pointed to the failure of our postmodern society to appreciate the psychological value of rites and rituals (Campbell, 1988; Rohr, 2011b; Le Grice, 2013). Stories of young people “coming-of-age” are common in mythic hero tales about children “awakening to the new world that opens at adolescence” (Campbell, 1988, p. 167). The hero’s journey “helps us pass through and deal with the various stages of life from birth to death” (Campbell, 1991, p. 56).

The second function of heroic transformation is that it promotes healing. Allison and Goethals (2016) argue that sharing stories about hero transformations can offer many of the same benefits as group therapy (Yalom and Leszcz, 2005). These benefits include the promotion of hope; the benefit of knowing that others share one’s emotional experiences; the fostering of self-awareness; the relief of stress; and the development of a sense of meaning about life. A growing number of clinical psychologists invoke hero transformations to help their clients acquire the heroic attributes of strength, resilience, and courage (Grace, 2016). Recent research on *post-traumatic growth* demonstrates that people can overcome severe trauma and even use it to transform themselves into stronger, healthier persons than they were before the trauma (Ramos and Leal, 2013).

The third function of transformations focuses on their ability to promote social unity.

According to Campbell (1972, p. 57), hero transformations “drop or lift [heroes] out of themselves, so that their conduct is not their own but of the species, the society.” The transformed hero is “selfless, boundless, without ego.” The most meaningful transformations are a journey from egocentricity to sociocentricity, from elitism to egalitarianism (Campbell, 1949; Wilber, 2007a,b; Rohr, 2011b). No longer psychologically isolated from the world, the transformed person enjoys a sense of communion with others. In his description of the hero’s journey, Campbell (1949, p. 25) wrote, “where we had thought to be alone, we *shall be with* all the world.” Friedman (2017) has introduced the construct of self-expansiveness describing how boundaries between ourselves and others, and even between ourselves and the world, can be seen as permeable. As Friedman puts it, “viewing others as an alternate manifestation of oneself can promote heroism, as one’s individual life is not viewed as separate” (p. 15).

Fourth, transformations also advance society in meaningful ways. The apex of the hero’s journey is the hero’s boon, or gift, to society. It is this gift that separates the hero’s journey from simply being a test of personal survival. For the quest to be heroic, the classic heroic protagonist must put her newly acquired insights and gifts to use in order to better the world (Campbell, 1949; Rohr, 2011b). The heroic boon to society follows the successful completion of the individual journey, and so we can say that the social boon is entirely dependent upon the hero’s personal transformation that made the individual quest a success. Hero mythology, according to Campbell (1972, p. 48), is designed to teach us that society is not a “perfectly static organization” but represents a “movement of the species forward.”

Finally, transformations contribute to a deepening of our spiritual and cosmic understanding of the universe. According to Campbell (1988, p. 152), the hero’s transformation involves learning “to experience the supernormal range of human spiritual life.” Myths, he said, “bring us into a level of consciousness that is spiritual” (p. 19). In every hero tale, the hero must “die spiritually” and then be “reborn to a larger way of living” (p. 141), a process that is the enactment of a universal spiritual theme of death being the necessary experience for producing new life (Campbell, 1991, p. 102). Hero transformations supply cosmic wisdom. van Gennep (1909) observed that transformative rituals in early human tribes have “been linked to the celestial passages, the revolutions of the planets, and the phases of the moon. It is indeed a cosmic conception that relates the stages of human existence to those of plant and animal life and, by a sort of pre- scientific divination, joins them to the great rhythms of the universe” (p. 194).

## Internal and External Sources of Transformation

Allison and Goethals (2017) distinguished between sources of transformative change that come from within the individual and sources that originate from outside the individual. There are several types of *internal* sources of transformation. For example, transformation can arise as a result of natural human development. An initial transformative event, a sperm cell fertilizing an egg, leads to a zygote transforming into an embryo, which then becomes a fetus, a baby, a toddler, a child, an adolescent, a young adult, a mid-life adult, and an elderly adult. Another internal source of change resides in people’s needs and goals. According to Maslow’s (1943) pyramid of needs, an individual is motivated to fulfill the needs at a particular level once lower level needs are satisfied. Once the needs at the four lower levels are satisfied, one is no longer concerned with them or driven by them. In effect, one transitions to higher levels and eventually achieves self-actualization, during which one might enjoy *peak experiences* of having discovered meaning, beauty, truth, and a sense of oneness with the world – a transformative state reminiscent of James’ (1902) description of the religiously converted individual.

A third internal source of transformative change is human transgression and failure. People often undergo significant change after being humbled by their “fallings and failings” (Rohr, 2011b, p. xv). Campbell (2004, p. 133) cautioned that not all heroic quests conclude with heroic triumph. “There is always the possibility for a fiasco,” he said. These occasional fiascos can inspire heroic transformations by producing the kind of suffering needed as impetus for a greater hero journey. It is a general truth that for substance abusers to be sufficiently motivated to seek recovery from their addictions, they must reach a profound level of pain and suffering, commonly referred to as “hitting rock bottom.” Suffering, according to Rohr (2011b, p. 68), “doesn’t accomplish anything tangible but creates space for learning and love.” This space has been called *liminal space* (Turner, 1966; van Gennep, 1909), defined as the transitional time and space between one state of being and an entirely different state of being. In liminal space, one has been stripped of one’s previous life, humbled, and silenced.

Transgressions, and the liminal space that follows them, are the fertile soil from which heroic transformations bloom.

Another internal source of transformation is what Allison and Goethals (2017) call an enlightened dawning of responsibility. This dawning is captured in a simple phrase, composed of 10 two-letter words, “If it is to be, it is up to me” (Phipps, 2011). There is a long history of social psychological work devoted to studying the forces at work that promote the dawning of responsibility in emergency settings (Latane and Darley, 1969). Research has shown that in a crisis a small but courageous minority of people do step up to do the right thing even when there are strong pressures to avoid assuming responsibility. These fearless social aberrants, most of whom are ordinary citizens, are able to transcend their circumstances and transform from ordinary to extraordinary. For example, about one-third of the participants in Milgram’s (1963) obedience study defied the authority’s command to continue applying painful electric shocks to another participant. Whistleblowers are another notable example; they have the mettle to step up and do right thing at great potential cost to themselves (Brown, 2017). Bystander training is now available to cultivate this dawning of responsibility in situations where transformative leadership is needed (Heroic Imagination Project, 2018).

External situational forces can also evoke transformative change. Situations, for example, can trigger emotional responses that transform us. This idea is consistent with the wisdom of James (1902, p. 77), who observed that “emotional occasions……are extremely potent in precipitating mental rearrangements.” Emotions need not be negative to induce change. Feelings of *elevation* can transform people psychologically and behaviorally (Haidt, 2003). People become elevated after witnessing a morally beautiful act, and this elevated feeling has been shown to produce altruistic acts (Thomson and Siegel, 2013). A second external source of transformation is the series of trials that all heroes must undergo during their journey. Suffering can be an internal cause of transformation when it results from self-destructive actions, but suffering caused by outside forces can serve as an external source of transformation. Campbell (1988, p. 154) argued that “trials are designed to see to it that the intending hero should be really a hero. Is he really a match for this task?” The time of greatest peril for the hero occurs when she enters the *belly of the whale* (Campbell, 1949). In stories of Jonah and Pinocchio, the belly can be entered literally, but typically the belly is symbolic of the hero’s deepest inner-demons which must be “disempowered, overcome, and controlled” (p. 180). According to Campbell (1988), the hero’s journey consists of the psychological task of overcoming one’s fears and slaying one’s dragons. This transformative process has been explored by positive psychologists who refer to it as part of the journey of post-traumatic growth, during which people are able to transform tragedy into triumph (Rendon, 2015).

A third external source of transformation is the vast hero literature and mythology to which we are exposed throughout our lives. Allison and Goethals (2014, 2016, 2017) have long argued that narratives about heroes, pervasive in all of storytelling from Gilgamesh to the present day, serve as a nourishing catalyst for transformative change. The central premise of the *heroic leadership dynamic* (HLD) is that our consumption of heroic tales takes place within an interactive system that is energetically in motion, and drawing us toward rising heroes and repelling us from falling ones. The HLD framework proposes two transformative functions of hero stories: an *epistemic* function and an *energizing* function. Hero narratives supply epistemic growth by offering mental schemas that describe prosocial action, reveal basic truths about human existence, unpack life paradoxes, and cultivate emotional intelligence. The epistemic value of hero tales is revealed in Campbell’s (1988) observation that hero mythology offers insights into “what can be known but not told” (p. 206) and that “mythology is the womb of mankind’s initiation to life and death” (Campbell, 2002, p. 34). Hero tales also offer energizing benefits, providing people with agency and efficacy. Narratives of heroism bring about moral elevation, repair psychic wounds, and promote psychological growth (Kinsella et al., 2015, 2017; Allison and Goethals, 2016).

The fourth external source of transformation is the social environment of the hero. In hero narratives and classic mythology, the hero’s journey is populated by numerous friends, companions, lovers, parent figures, and mentors who assist the hero on her quest (Campbell, 1949). The hero is always helped along the journey by the actual, imagined, or implied presence of others. Campbell also discussed the importance of encounters with parental figures; male heroes seek atonement with father figures, and female heroes seek it with mother figures.

Campbell also described the hero’s brush with lovers and temptresses, who can either assist, distract, or do harm to the hero. Most people who are asked to identify their heroes describe a mentor or coach who exerted a transformative effect on them (Allison and Goethals, 2011; Goethals and Allison, 2012, 2014).

Campbell (1949) argued that the appearance of a mentor during the initiation stage of the hero’s journey is a critically important component of the quest. Mentors help heroes become transformed, and later, having succeeded on their journeys, these transformed heroes then assume the role of mentor for others who are at earlier stages of their quests. In short, “transformed people transform people” (Rohr, 2014, p. 263). Mentors can have a transformative effect with their words of advice, with their actions, or both. Words can fall on deaf ears but one’s actions, attitudes, and lifestyle can leave a lasting imprint. St. Francis of Assisi expressed it this way: “You must preach the Gospel at all times, and when necessary use words” (Rohr, 2014, p. 263). A mentor can be viewed as a type of hero who enhances the lives of others (Kinsella et al., 2015).

The hero’s journey offers a transformative experience toward wisdom that can be shared later with others. In short, the journey prepares people for leadership roles. According to Burns (1978), transforming leaders strive to satisfy followers’ lower needs (e.g., survival and safety) in preparation for elevating them to work together to produce significant higher-level changes. Burns portrayed transforming leadership as collaborative engagement “in such a way that leaders and followers raise one another to higher levels of motivation and morality” (p. 20). Followers are thus “elevated,” creating a “new cadre of leaders” (p. 20). This conceptualization is consistent with Campbell’s (1949) emphasis on the role of mentorship during the hero’s journey. The mentor elevates the hero and prepares her for her future role as a mentor to others. Burns’ framework also makes explicit a notion that is largely implicit in Maslow’s (1943) model, namely, that the self-actualized person has become an elder, a mentor figure, and a moral actor who wields transformative influence over others. Erikson’s (1994) theory of lifelong development makes the similar claim that older *generative* individuals, having been given so much early in life, are now in a position to give back to younger people.

Other theories also point to the transformative effect of mentoring and leadership.

Hollander (1995) proposed a two-way influence relationship between a leader and followers aimed primarily at attaining mutual goals. Hollander defined leadership as “a shared experience, a voyage through time” with the leader in partnership with followers to pursue common interests.

For Hollander, “a major component of the leader–follower relationship is the leader’s perception of his or herself relative to followers, and how they in turn perceive the leader” (p. 55). Tyler and Lind (1992) have shown that these perceptions are crucially important in cementing good follower loyalty. Followers will perceive a leader as a “legitimate” authority when she adheres to basic principles of procedural justice. Leaders who show fairness, respect, and concern for the needs of followers are able to build followers’ self-esteem, a central step in Maslow’s (1943) pyramid, thereby fostering followers’ transformative movement toward meeting higher-level needs.

## Three Transformative Arcs of Heroism

Allison and Goethals (2017) identified three deficits of the hero at the initial stage of her journey. The untransformed hero is lacking (1) a sociocentric view of life; (2) an autonomy from societal norms that discourage transformation; and (3) a mindset of growth and change. Below we explain how the arc of heroic metamorphosis bends toward sociocentricity, autonomy, and growth.

### Egocentricity to Sociocentricity

Campbell (2004, p. 55) believed that one of the central functions of hero mythology is to “get a sense of everything – yourself, your society, the universe, and the mystery beyond – as one great unit.” He claimed that “when we quit thinking primarily about ourselves and our own self-preservation, we undergo a truly heroic transformation of consciousness” (Campbell, 1988, p. 155). In most hero narratives, the hero begins the journey disconnected from the world. She is a self-centered, prideful individual whose sole preoccupation is establishing her identity, her career, and her material world. The entire point of her hero journey is to awaken her to the broader goal of thinking beyond herself in achieving communion with the entire world and universe (Friedman, 2017). To the extent that we spend the first stages of our lives selfishly building our personal identities and careers, we may be designed to awaken in later stages to our original predisposition toward sociocentricity (Rohr, 2011b). Campbell (2001) urged us all to cultivate this greater purpose of forming compassionate unification with all of humanity. He believed this awakening is the central function of hero mythology.

### Dependency to Autonomy

A person’s willingness to deviate from the dominant cultural pattern is essential for heroic transformation. Heroes do the right thing, and do what they must do, regardless of authority, tradition, and consequence. Maslow (1943) called this characteristic *autonomy*. “There are the ‘strong’ people,” wrote Maslow, “who can easily weather disagreement or opposition, who can swim against the stream of public opinion and who can stand up for the truth at great personal cost” (p. 379). Fulfillment of the lower needs in the pyramid is essential for autonomy to develop in individuals. “People who have been made secure and strong in the earliest years tend to remain secure and strong thereafter in the face of whatever threatens” (p. 380). Zimbardo (2008) has championed the idea that heroes are people with the ability to resist social pressures that promote evil, and that such resistance requires the moral courage to be guided by one’s heart rather than by social cues. Zimbardo and other hero activists drive home the point that “the opposite of a hero isn’t a villain; it’s a bystander” (Chakrabortty, 2010; Langdon, 2018). While the transformed hero enjoys “union with the world,” she remains an autonomous individual who can establish her own path in the world that is unfettered by pressures to conform to social pressures.

### Stagnation to Growth

One can be autonomous but not necessarily growing and stretching toward realizing one’s full potential. The pre-transformed hero naturally resists change, and thus severe setbacks may be her only impetus to budge. Without a prod, she will remain comfortable in her stagnation, oblivious to the idea that anything needs changing. The hero’s journey marks the death of pretense and inauthenticity, and the birth of the person one is meant to be. Campbell (1988, p. 168) described the process as “killing the infantile ego and bringing forth an adult.” Sperry (2011) has argued that people are so attached to their false selves that they fear the death of the false self even more than they fear the death of their physical self. Our growth can also be inhibited by a phenomenon called the crab bucket syndrome (Simmons, 2012). This syndrome describes the consequences of our entrenchment with our families, our friends, and our communities, and they with us. Any attempt we make to crawl up and out of the bucket is met with failure as the crabs below us pull us back down. For most of us, the hero’s journey represents the best way, and perhaps the only way, to escape the bucket and discover our true selves. Campbell (1991) argued that a healthy, transformed individual accepts and embraces her growth and contradictions. “The psychological transformation,” wrote Campbell, “would be that whatever was formerly endured is now known, loved, and served” (p. 207).

## Three Activities Promoting Transformation

Can anything be done to promote heroic transformation? We noted earlier that one cannot be in charge of one’s own heroic transformation. According to Rohr (2011a), engineering our own personal metamorphosis on our own “is by definition not transformation. If we try to change our ego with the help of our ego, we only have a better-disguised ego” (p. 5). There are things we can do, however, to make transformation more likely. From our review of theory and research on heroism, developmental processes, leadership, and spiritual growth, we can identify three broad categories of activities that encourage transformation. These activities include participation in training and developmental programs, spiritual practices, and (of course) the hero’s journey. On the surface these activities appear dissimilar, yet engaging in these practices produces similar transformative results.

### Training and Development Practices

In examining the characteristics of people who risked their lives to save others, Kohen et al. (2017) discovered several important commonalities. They found that these heroes “imagined situations where help was needed and considered how they would act; they had an expansive sense of empathy, not simply with those who might be considered ‘like them’ but also those who might be thought of as ‘other’ in some decisive respect; they regularly took action to help people, often in small ways; and they had some experience or skill that made them confident about undertaking the heroic action in question” (p. 1). With this observation, Kohen et al. (2017) raise four points about preparation for heroism. First, they note the importance of imagining oneself as ready and capable of heroic action when it is needed. This imagination component involves the development of mental scripts for helping, an idea central to Zimbardo’s Heroic Imagination Project (2018) hero training programs. Established a decade ago, the Heroic Imagination Project aims to encourage people to envision themselves as heroes and to “prepare heroes in training for everyday heroic action.” The group achieves this goal by training ordinary people to “master social and situational forces as well as their automatic human tendencies in order to act in ways that are kind, prosocial, and even heroic.” Participants are trained to improve their situational awareness, leadership skills, moral courage, and sense of efficacy in situations that require action to save or improve lives.

Second, Kohen et al. (2017) emphasize the importance of empathy, observing that heroes show empathic concern for both similar and dissimilar others. A growing body of research supports the idea that empathy can be enhanced through training, an idea corroborated by the proliferation of empathy training programs around the world (Tenney, 2017). Svoboda (2013) even argues that empathy and compassion are muscles that can be strengthened with repeated use. Third, Kohen et al. (2017) note that heroes regularly take action to help people, often in small ways. Doing so may promote the self-perception that one has heroic attributes, thereby increasing one’s chances of intervening when a true emergency arises. Finally, Kohen et al. (2017) observe that heroes often have either formal or informal training in saving lives. These skills and experiences may be acquired from training for the military, law enforcement, or firefighting, or they may derive from emergency medical training, lifeguard training, and CPR classes (Svoboda, 2013).

In a similar vein, Kramer (2017) has devised a methodology for helping people develop the courage to pursue their most heroic dreams and aspirations in life. He identifies such courage as *existential courage*, consisting of people’s identity aspirations and strivings for their lives to feel meaningful and consequential. Kramer’s technique involves fostering people’s willingness to take psychological and social risks in the pursuit of desired but challenging future identities. His “identity lab” is a setting where students work individually and collaboratively to (1) identify and research their desired future identities, (2) develop an inventory or assessment of identity- relevant attributes that support the realization of those desired future identities, (3) design behavioral experiments to explore and further develop those self-selected identity attributes, and, finally, (4) consolidate their learnings from their experiments through reflection and assessment. Kramer’s results show that his participants feel significantly more “powerful,” “transformative,” “impactful,” and “effective” in pursuing their identity aspirations. They also report increased self-efficacy and resilience.

Another example of training practices can be found in initial rituals and rites of passages found in many cultures throughout the world. Although modern Western cultures have eliminated the majority of these practices, most cultures throughout history did deem it necessary to require adolescents, particularly boys, to undergo rituals that signaled their transformation into maturity and adulthood (Turner, 1966; van Gennep, 1909). In many African and Australian tribes, initiation requires initiates to experience pain, often involving circumcision or genital mutilation, and it is also not uncommon for rituals to include a challenging survival test in nature. These initiation tests are considered necessary for individuals to become full members of the tribe, allowing them participate in ceremonies or social rituals such as marriage. Initiations are often culminated with large elaborate ceremonies for adolescents to be recognized publicly as full-fledged adult members of their society.

Child-rearing can serve as another type of transformative training practice. A striking example can be seen in Fagin-Jones’s (2017) research on how parents raised the rescuers of Jews during the Holocaust. Fagin-Jones found that the parenting practices of rescuers differed significantly from the parenting of passive bystanders. Rescuers reported having loving, supportive relationships with parents, whereas bystanders reported relationships with parents as cold, negative, and avoidant. More rescuers than bystanders recalled their parents as affectionate and engaged in praising, hugging, kissing, joking, and smiling. These early cohesive family bonds encouraged other-oriented relationships based on tolerance, inclusion, and openness.

Rescuers reported that their family unit engendered traits of independence, potency, risk-taking, decisiveness, and tolerance. Bystanders, in contrast, recalled a lack of familial closeness that engendered impotence, indecisiveness, and passivity. Rescuers’ parents were less likely than bystanders’ parents to express negative Jewish stereotypes such as “dishonest,” “untrustworthy,” and “too powerful.” Overall, rescuers were raised to practice involvement in community, commitment to others’ welfare, and responsibility for the greater good. In contrast, bystanders’ parents assigned demonic qualities to Jews and promoted the idea that Jews deserved their fate.

### Spiritual Practices

For millennia, spiritual gurus have extolled the benefits of engaging in a variety of spiritual practices aimed at improving one’s mental and emotional states. Recent research findings in cognitive neuroscience and positive psychology are now beginning to corroborate these benefits. Mindfulness in particular has attracted widespread popularity as well as considerable research about its implications for mental health. The key component of mindfulness as a mental state is its emphasis on focusing one’s awareness solely on the present moment. People who practice mindful meditation show significant decreases in stress, better coping skills, less depression, improved emotional regulation, and higher levels of resilience (Hofmann et al., 2010). Mindful meditation quiets the mind and thus “wakes us up to what is happening,” allowing “contact with life” (Hanh, 1999, p. 81). Tolle (2005) argues that living in the present moment is a transformative experience avoided by most people because they habitually choose to clutter their minds with regrets about the past or fears about the future. He claims that “our entire life only happens in this moment. The present moment is life itself” (p. 99). Basking in the present moment is the basis of the psychological phenomenon of “flow” described by Csikszentmihalyi (2008). When experiencing flow, people are “in the zone,” fully present, and completely “immersed in a feeling of energized focus” (p. 45).

Related to mindfulness is the process of non-dualistic thinking (Loy, 1988) also called *right* thinking (Hanh, 1999), *contemplative* thinking (Rohr, 2009), *third-force* thinking, and the third eye (Song, 2002). The Indian “tika” placed on the human forehead is more than decoration; it signifies a non-dual way of viewing the world. According to Rohr (2009), non-dual thinking is deemed necessary for understanding phenomena that defy rational analysis: love, death, God, suffering, and eternity. The transcendent nature of mindful, non-dual thinking shares many of the characteristics of the heroically transformed mind that we have discussed in this article.

The spiritual attribute of humility can also be transformative. When asked to name four cardinal virtues, St. Bernard is reported to have answered: “Humility, humility, humility, and humility” (Kurtz and Ketcham, 1992). Humility has been shown to be linked to increased altruism, forgiveness, generosity, and self-control (Worthington et al., 2017). One can argue that humility cannot be practiced, as the idea of getting better at humility runs contrary to being humble. However, we suspect that one can practice humility by adopting the habit of admitting mistakes, acknowledging personal faults, avoiding bragging, and being generous in assigning credit to others.

Gratitude is another transformative spiritual practice validated by recent research. Algoe (2012) found that gratitude improves sleep, patience, depression, energy, optimism, and relationship quality. Practitioners have developed gratitude therapy as a way of helping clients become happier, more agreeable, more open, and less neurotic. Moreover, neuroscientists have found that gratitude is associated with activity in areas of the brain associated with morality, reward, and value judgment (Emmons and Stern, 2013). Closely related to gratitude are experiences with wonder and awe, which have been shown to increase generosity and a greater sense of connection with the world (Piff et al., 2015). Enjoying regular doses of wonder is a telltale trait of the self-actualized individual (Maslow, 1943).

Another transformative spiritual practice is forgiveness. Research shows that people who are able to forgive others have improved relationships, better mental health, lower stress and hostility, improved blood pressure, less depression, and a healthier immune system (Worthington, 2013). “Letting go” is another spiritual practice that can produce transformation. It has also been called release, acceptance, or surrender. Buddhist teach Hanh (1999, p. 78) claims that “letting go give us freedom, and freedom is the only condition for happiness.” James (1902) also described the beneficial practice of letting go among religiously converted individuals: “Give up the feeling of responsibility, let go your hold, resign the care of your destiny to higher powers, be genuinely indifferent as to what becomes of it all, and you will find not only that you gain a perfect inward relief, but often also, in addition, the particular goods you sincerely thought you were renouncing” (p. 110).

Finally, we turn to the complex emotion of love as a transformative agent. In addition to starring in *Casablanca*, Humphrey Bogart played the lead role in *Sabrina*, another film demonstrating the transformative power of love. In *Sabrina*, Bogart played the role of Linus, a workaholic CEO who has no time for love. His underachieving brother David begins a romance with a young woman named Sabrina, and it becomes clear that this budding relationship jeopardizes a multi-million-dollar deal that the company is about to consummate. To undermine the relationship, Linus pretends to show romantic interest in Sabrina, and he succeeds in winning her heart. Despite the pretense, Linus falls in love for the first time in his life, resigns as CEO, and runs away with Sabrina to Paris. Love has completely transformed him from a cold, greedy businessman into a warm, enlightened individual. Similar transformations in film and literature are seen in Ebenezer Scrooge (in *A Christmas Carol*), the Grinch (in *How the Grinch Stole Christmas*), Phil Connors (in *Groundhog Day*), and George Banks (in *Mary Poppins*).

In *Man’s Search for Meaning*, Frankl (1946, p. 37) wrote, “The salvation of man is through love and in love.” Hanh (1999, p. 170), moreover, weighs in that “love, compassion, joy, and equanimity are the very nature of an enlightened person.” Loving kindness also transforms us biologically (Keltner, 2009). People who make kindness a habit have significantly lower levels of stress hormones such as cortisol. Making an effort to help others can lead to decreased levels of anxiety in individuals who normally avoid social situations. Being kind and even witnessing kindness have also been found to increase levels of oxytocin, a hormone associated with lower blood pressure, more sound sleep, and reduced cravings for drugs such as alcohol and cocaine. Loving others lights up the motivation and reward circuits of the limbic system in the brain (Esch and Stefano, 2011). Research also reveals that people who routinely show acts of love live longer compared to people who perform fewer loving actions (Vaillant, 2012).

### The Hero’s Journey

We opened this article by noting that the only way most of us undergo transformation is to embark on the hero’s journey. While we have complete control over whether we receive training that can facilitate a heroic metamorphosis, and over whether we engage in spiritual practices, we have far less control over our participation in the classic hero’s journey. We can only remain open and receptive to the ride that awaits us. As we have noted, our departure on the journey can be jarring – we often experience an accident, illness, transgression, death, divorce, or disaster. The best we can do is fasten our seatbelts and trust that the darkness of our lot will eventually transform into lightness. But we cannot remain passive. During the journey we must be diligent in doing our part to secure allies and mentors, and to take actions that cultivate strengths such as resilience, courage, and resourcefulness (Williams, 2018). After being transformed ourselves, we feel the obligation to transform others in the role of mentor. Having traversed the heroic path, we may use our heroism to craft a newfound purpose for our existence, a purpose that drives us to spend our remaining years making a positive difference in people’s lives. Bronk and Riches (2017) call this process *heroism-guided purpose*.

## Additional Issues Worth Pondering

Several unexplored issues involving heroic transformation deserve more thorough treatment than we can devote to them here. These issues focus on education, religion, gender, inclusive transcendence, and barriers to transformation. We give brief attention to these topics below.

### Education and Transformation

On July 16, 2003, legendary President of South Africa Nelson Mandela delivered a speech in support of the Mindset Network, a non-profit organization designed to improve educational opportunities for children of all ages. “Education,” he said, “is the most powerful weapon which you can use to change the world.” This statement attracted widespread media attention and remains a highly recurring internet meme today. A Google search of “education can change the world” yields thousands of hits echoing Mandela’s claim and extending the idea to include education being the key “to success,” “to happiness,” “to freedom,” “to the world,” and “to the future.” Summing up our supreme collective confidence in education, United Nations Emergency Relief Coordinator Mark Lowcock declared that “Education is the key to everything” (Theirworld, 2017).

Are these claims true? We believe it is a mistake and perhaps even dangerous to equate education with transformation. Consider, for example, the link between education and crime. Some studies suggest that education mitigates crime (Buonanno and Leonida, 2006; Machin et al., 2011) while other studies find that education either plays little or no role in preventing violence. Bergen and Pandey (2005) report that the vast majority of terrorists who perform violent acts are college educated. For example, all 12 men involved in the 1993 World Trade Center attack had a college education. All the pilots in the September 11^th^ terrorist attacks and their collaborators, as identified by the 9/11 commission, attended universities. The lead pilot, Mohamed Atta, was college-educated, and the operational planner, Khalid Sheikh Mohammed, studied engineering in North Carolina. The chilling masked figure on many ISIS beheading videos was Mohammed Emwazi, who had a college degree in computer programming. In the same vein, Ramsland (2015) has found that some of the most notorious serial killers of our time were highly educated, including Ted Bundy and the “Unabomber” Ted Kaczynski.

We do not wish to undersell education’s positive consequences for individuals and societies. Improving educational opportunities for citizens no doubt helps people satisfy needs in Maslow’s (1943) hierarchy, especially those at the lower levels of the pyramid. Nelson Mandela was no doubt correct about education improving the quality of life for communities operating near subsistence levels. Our claim is that education is insufficient for meeting higher level needs of esteem and for cultivating social belongingness, self-transcendence, union with the world, and self-actualization. In short, education is a beginning step toward transformation but falls short in fully producing a truly awakened individual.

### Religion and Transformation

As noted earlier, James (1902) described the psychological consequences of religious conversion as including feelings of peace, the ability to see clearly, the sense of union with all of humanity, a feeling of newness, the experience of happiness, the desire for generosity, and the sense of being part of something bigger than oneself. While these results of conversion are all signs of healthy religion, many of us are very well aware of “religious” individuals who preach war instead of peace, who exclude rather than include, who display anger in lieu of joy, and who show greed instead of generosity. In short, being “religious” and even engaging in religious practices such as attending church does not guarantee the kind of religious conversion experiences described by James. In fact, going through the motions of religion can heighten one’s sense of righteousness and arrogance, setting in motion a dark transformation toward principles that are antithetical to James’ observation of mature religion. Many people who are “holier than thou” end up holier than no one. Rohr (2010) argues that the litmus test for healthy spiritual transformation is whether one shows “a movement toward the edge, the outside, the lower, the suffering, and the simple. It’s never about climbing.”

### Women as Transformers

In his studies of initiation rituals worldwide, Rohr (2005) observed that non-western cultures throughout history have been more likely to require males to participate in these rites of passage as compared to females. Underlying this gender difference is the widespread belief that young males require initiation rituals to transform them into men, whereas young females tend to be naturally capable of transforming into womanhood without formal rituals. Differences in biology and culturally assigned gender roles have been posited to explain this difference (Rohr, 2005; Formica, 2009). For women, transformation is corporeal. Women personally undergo biological transformations in processes such as menstruation, pregnancy, labor, and breastfeeding. Throughout most of human history, women have also been assigned culturally mandated activities involving transformation. For example, child-rearing traditionally involved women transforming children into adults. Moreover, most human cultures have historically assigned women the task of preparing food for the family, during which women transformed wheat into bread and cream into butter.

If, as we have argued, transformation involves promoting unity and adopting a sociocentric mindset, then women may be agents of transformation. Throughout history, men have built things, fixed things, and defended us from things (Rohr, 2005) – all in the service of satisfying lower level needs. True transformation, however, occurs at higher levels where women may have the advantage. Rohr has even boldly claimed that “transformation is deeply embedded in feminine consciousness” (see also Ross, 2017). In her review of research on gender differences in leadership effectiveness, Hoyt (2014) found convincing evidence that women may be more transformative as political leaders. Compared to men, women leaders are more likely to improve standards of living, education, and healthcare. They enjoy more success in peace negotiations and are more likely to reach across party lines. Women more so than men are likely to adopt democratic and participatory styles of leadership. Moreover, women are more likely to follow ethical guidelines, engage in philanthropy, and promote the welfare of women, children, and families. With all their accomplishments as leaders, women may also show more humility than men (Fumham et al., 2001; Perry, 2017). Over 2500 years ago, the Tao Te Ching offered this wise description of women as humble, transformative leaders:

Can you play the role of woman?

Understanding and being open to all things…

Giving birth and nourishing,

Bearing but not possessing,

Working yet not taking credit,

Leading yet not dominating,

This is the Primal Virtue.

### Transcend and Include

Central to the phenomenon of transformation is the principle of *transcend and include* (Wilber, 2001). Higher stages of transformation do not discard the values of the lower stages; they include them. When we are young, we hold strong opinions that later seem naïve to us, yet we are not necessarily “wrong” at the time; we are merely incomplete. An illustration of this idea can be found in our musings about our childhood baseball heroes, Willie Mays (for George Goethals) and Willie Stargell (for Scott Allison). We both freely admit that our taste in heroes has evolved and matured since the 1950s and 1960s, yet if you ask us if that means that Mays and Stargell are no longer our heroes, we will quickly tell you that they remain our heroes to this very day. Maintaining this preference exemplifies the principle of transcend and include.

Transformation to a higher level of consciousness always transcends but also includes the lower levels (Rohr, 2011b). This does not mean that we equate Mays and Stargell with Gandhi and Mandela. It means that we appreciate their heroic influence on us during a crucial time in our development.

Campbell’s (1949) understanding of the transform and include principle is seen in his description of the transformed hero as the “master of both worlds.” At the end of their transformative journey, heroes are as comfortable navigating in their original world as in the new world that they now inhabit. There are implications of this principle for gender roles. Male- oriented activities of making, fixing, and protecting must be transcended by female-oriented activities of inclusion, participation, and harmony. But with transcendence must come inclusion, as we cannot expect to survive as a society without always leaving room for those so-called male activities.

### Transformation Toward Psychopathology

Heroic transformation does not always lead to improvement in an individual’s well-being. Recent research has revealed that adopting a heroic self-concept can at times produce significant psychological maladjustment (Shahar, 2013; Israeli et al., 2018). From this perspective, a heroic self-representation may develop when people experience personal threat, stress, and challenge, either in themselves or in others to whom they are close. These heroic self-representations can assume the form as the *self-as-savior*, the *self-as-conqueror*, or *heroic identification*. When confronting these psychological challenges, people may identify with the ideal heroic image of the person who can conquer any difficult obstacle or who can heroically remove those obstacles for suffering others. The consequences of taking on this role of a hero can be significant increases in perceived stress, self-criticism, lack of a sense of coherence, general psychopathology, maternal overprotection, dissociative depersonalization and absorption, transliminality, PTSD severity, and attachment anxiety.

Shahar (2013) and Israeli et al. (2018) have uncovered convincing evidence for this type of pathological heroic transformation. These scholars studied adults during a prolonged exposure ‘Operation Protective Edge,’ which occurred in Israel between July 8, 2014 and August 26, 2014 (Israeli et al., 2018). The operation measured Israeli citizens’ emotional states while they were exposed to extensive air strikes, ground fighting in Gaza, and continuous large-scale rocket fire from Gaza to Israel. The results showed that participants’ heroic identification predicted increased anxious mood and negative affect. Moreover, participants who viewed themselves as self-as-savior showed an increased anxious mood under high levels of perceived-stress related to the missile attacks. Israeli et al. (2018, p. 23) concluded that “under stress, heroic identification increases characterological self-blame/self-criticism and experiential avoidance, and decreases help-seeking.” These findings are fascinating in pointing toward the potential harm associated with undergoing a heroic transformation. Whereas we argue that heroic transformation is a necessary and positive step toward mature growth and achieving one’s full potential, it seems clear that taking one’s heroism to an extreme under stressful circumstances can lead to psychological harm. We believe that the research reported by Shahar (2013) and Israeli et al. (2018) is extremely important in identifying boundary or delimiting conditions of positive heroic transformation effects. Future research might productively be directed toward further establishing the circumstances under which adopting a heroic self-representation yields favorable versus unfavorable consequences for people.

### Barriers to Transformation

We now turn to factors that can stand in the way of people undergoing a positive transformative experience in life. The largest barrier, of course, is a person’s unwillingness to heed the call to go on the hero’s journey. We all know people, including prominent world leaders, who are “stuck” in early stages of development. It would behoove the world to understand why so many people are stuck and what can be done to nudge more of us along the transformative journey. Earlier we reviewed activities that promote transformation, and one might argue that any barriers to change are merely the inverse of these promotional activities. While there may be some truth in this idea, it is also true that some barriers are less intuitive or obvious than one might suspect. The great Islamic poet Rumi once offered this advice to those seeking enlightenment: the task is sometimes not to pursue a transformative loving experience “but merely to seek and find all the barriers within yourself that you have built against it” (Barks, 2005, p. 18).

A major source of arrested development is the problem of self-ignorance. A recurring theme in psychological research is that people are unaware of much of their own psychological functioning (Nisbett and Wilson, 1977; Wegner, 2002; Bargh and Morsella, 2008; Alicke, 2017). This lack of self-awareness may explain people’s resistance to transformative growth. Early psychoanalytic theories of Freud, Adler, and Horney were the first to point to the destructive effects of behaving unconsciously. Jung (1956) described the *shadow* as the dark, unknown aspects of our personalities that prevent us from transforming into our full potential. Building on Jung’s work, Campbell observed that all “the images of [hero] mythology are referring to something in you,” and that our shadow impedes our ability to make the best use of these images (p. 68).

A second barrier is found in impoverished environments that deny people opportunities for transformation. Maslow’s (1943) model of hierarchical needs suggests that people can get stuck at lower stages of the hierarchy that focus on satisfying basic biological and security needs. Heroic potential may be suppressed when individuals are afflicted by poverty or safety concerns that hinder their ability to progress upward in the hierarchy toward higher-level goals. Resolving this problem is easy in theory but extremely difficult in practice, as most world societies either lack the will or the means to eliminate poverty. Related to this idea is another barrier – exposure to traumatic events that can impede people’s ability to undergo transformative growth. Trauma disrupts people’s sense of safety and their ability to cope with the overwhelming threat and danger, damaging their physical, emotional, and cognitive functioning processes (Keck et al., 2017). Safety and security needs become paramount to the traumatized individual, rendering higher level needs unimportant. The good news is that most people can show great progress in recovering from the deleterious effects of trauma. This healing is the basis of the hopeful phenomenon of post-traumatic growth (Rendon, 2015).

A fourth barrier to transformation is people’s strong tendency to self-identify as victims.

Individuals who have been harmed and who derive their entire personal identity from being wronged by someone else, or by society, may find it difficult to grow and transcend their victimhood. We are not making the claim that there are no legitimate victims; there most certainly are people who have been harmed and have real grievances. Our argument is that adopting a strong and permanent victim identity is a sure way of avoiding growth and moving beyond the pain of having been harmed. A highly unfortunate consequence of harboring a victim mindset is the need to scapegoat. People tend to reason that if someone has harmed them, then that perpetrator must be punished. There is no doubt that scapegoating others has been the primary cause of most violence and warfare throughout human history. Until people learn to take individual responsibility for their lives and for their anger, the deadly duo of victimhood and scapegoating will continue to work in concert to thwart heroic transformation.

Another barrier to transformation lies in the absence of good mentorship. Social sources of wisdom, inspiration, and change are critical elements of the hero monomyth as described by Campbell (1949). These social sources appear in the form of friends, mentors, peers, and allies, all of whom represent rich and essential sources of transformation. There are times, moreover, when people encounter the wrong mentor whose advice does more harm than good. Allison and Smith (2015) used the term *dark mentors* to describe these damaging guides who not only undermine people’s ability to walk the heroic path; they encourage us down the *wrong* path.

Severe mental and physical illness can also impede people’s ability to undergo heroic transformation. Most individuals facing severe mental or physical disability are unable to reap the benefits of the hero’s journey because they are preoccupied with managing their condition. Related to this problem is the prevalence of narcissism. Psychologists believe that roughly 6% of US adults are afflicted with narcissistic personality disorder (Bressert, 2018), which means that at least 15 million Americans may be narcissists. The characteristics of narcissism are a heightened sense of importance, a drive for unlimited success, a belief in one’s special nature, exploitation of people, little empathy, and an arrogant attitude. Narcissists are unlikely to undergo heroic transformation because they don’t believe they need one and thus avoid it entirely (Worthington and Allison, 2018). The narcissist assigns blame for his problems to others, leading the him to believe that other people need to change rather than the narcissist himself.

Finally, people may avoid heroic transformation because they lack psychological flexibility, defined as an individual’s ability to adapt to fluctuating situational demands. Those classified as low in psychological flexibility have been shown to experience less growth and development (Kashdan and Rottenberg, 2010). To help people overcome inflexibility, Hayes et al. (2011) developed a therapeutic approach called acceptance and commitment therapy (ACT). The goal of ACT is to increase people’s ability to remain in the present moment as a conscious human being, and to learn new behaviors that serve desired goals. Psychological flexibility can be achieved through six core ACT processes, several of which sound like mindful pathways to Buddhist enlightenment. The six elements of ACT are acceptance, cognitive defusion, presence, seeing the self in context, values, and committed action. All of these processes reflect positive psychological and spiritual skills that enable people to grow and evolve into healthy adaptive human beings. They also resemble Franco et al. (2016) skillset of heroic eudaimonia, which includes mindfulness, autonomy, and efficacy (see also Jones, 2017).

## Conclusion

This article has reviewed the functions, processes, and consequences of the hero’s transformation. William James once observed, “Whenever one aim grows so stable as to expel definitively its previous rivals from an individual’s life, we tend to speak of the phenomenon, and even *wonder* at it, as a transformation” (James, 1902, p. 70, italics added). James’ use of the word “wonder” implies that people are moved by the transformations they see in people, and also that these transformations are a rare occurrence. As did James, we suspect that many people spend their entire lives resisting change, denying the need for it, and suffering as a result of avoiding it. As Jung (1945) observed, “There is no coming to consciousness without pain. People will do anything, no matter how absurd, in order to avoid facing their own soul. One does not become enlightened by imagining figures of light, but by making the darkness conscious” (p. 335).

The transformed hero exemplifies the zenith of human development. Psychologists have called this state self-actualization (Maslow, 1943), the condition of well-being that allows people to flourish (Seligman, 2011), the achievement of “bliss” (Campbell, 1988), and the experience eudaimonia (Franco et al., 2016). From their journey, heroes accumulate wisdom about their place in the world; they acquire the courage to face their deepest fears; they connect with all of humanity; they seek justice no matter the cost to themselves; they show humility; and they embark on a journey that “opens the world so that it becomes transparent to something that is beyond speech, beyond words, in short, to what we call transcendence” (Campbell, 2014, p. 40; see also Friedman, 2017). The wisdom of writers and philosophers, from Homer in 800 BCE to Phil Zimbardo today, informs us that we are all called to lead a heroic life. Yet most people are unaware of this fact, or they face impediments that impede the realization of their heroic potential. If the ultimate goal of the hero’s journey is for the hero to bestow the world with transformative gifts, then one would think that the world would be doing everything possible to promote the hero’s journey for everyone. We hope that this article represents progress toward shedding light on why transformation is elusive and what can be done to promote it.

## Author Contributions

All authors contributed equally to the development and expression of the ideas in this article.

## Conflict of Interest Statement

The authors declare that the research was conducted in the absence of any commercial or financial relationships that could be construed as a potential conflict of interest.
